# Induction of aquaporin 4-reactive antibodies in Lewis rats immunized with aquaporin 4 mimotopes

**DOI:** 10.1186/s40478-020-00920-x

**Published:** 2020-04-15

**Authors:** Irina Tsymala, Magdalini Nigritinou, Bleranda Zeka, Rouven Schulz, Felix Niederschick, Mia Matković, Isabel J. Bauer, Michael Szalay, Kathrin Schanda, Magdalena Lerch, Tatsuro Misu, Kazuo Fujihara, Jeffrey L. Bennett, Charlotte Dahle, Florence Pache, Paulus Rommer, Fritz Leutmezer, Zsolt Illes, Maria Isabel Leite, Jacqueline Palace, Petra Scholze, Markus Reindl, Hans Lassmann, Monika Bradl

**Affiliations:** 1 Department Neuroimmunology, Medical University Vienna, Center for Brain Research, Spitalgasse 4, A-1090 Vienna, Austria; 2 Department Pathobiology of the Nervous System, Medical University Vienna, Center for Brain Research, Spitalgasse 4, A-1090 Vienna, Austria; 3 Clinical Department of Neurology, Medical University of Innsbruck, Innrain 66/2, A-6020 Innsbruck, Austria; 4 Departments of Multiple Sclerosis Therapeutics and Neurology, Tohoku University Graduate School of Medicine, 1-1 Seiryomachi, Aobaku, Sendai, 980-8574 Japan; 5 Department of Neurology, Neuroscience Program, University of Colorado, Denver, CO 80045 USA; 6 Department of Clinical and Experimental Medicine, Faculty of Health Sciences, Linköping University, Linköping, Sweden; 7 Department of Neurology and NeuroCure Clinical Research Center, Charité-Universitätsmedizin Berlin, Berlin, Germany; 8 Department of Neurology, Medical University Vienna, Vienna, Austria; 9 Department of Neurology, Odense University Hospital and University of Southern Denmark, Odense, Denmark; 10 Nuffield Department of Clinical Neurosciences, John Radcliffe Hospital, Oxford, UK

**Keywords:** Neuromyelitis optica spectrum disorders, Antibodies, Mimotopes, Aquaporin 4, Infections, Animal model

## Abstract

Most cases of neuromyelitis optica spectrum disorders (NMOSD) harbor pathogenic autoantibodies against the water channel aquaporin 4 (AQP4). Binding of these antibodies to AQP4 on astrocytes initiates damage to these cells, which culminates in the formation of large tissue destructive lesions in the central nervous system (CNS). Consequently, untreated patients may become permanently blind or paralyzed. Studies on the induction and breakage of tolerance to AQP4 could be of great benefit for NMOSD patients. So far, however, all attempts to create suitable animal models by active sensitization have failed. We addressed this challenge and identified peptides, which mimic the conformational AQP4 epitopes recognized by pathogenic antibodies of NMOSD patients. Here we show that these mimotopes can induce the production of AQP4-reactive antibodies in Lewis rats. Hence, our results provide a conceptual framework for the formation of such antibodies in NMOSD patients, and aid to improve immunization strategies for the creation of animal models suitable for tolerance studies in this devastating disease.

## Introduction

Pathogenic autoantibodies directed against the water channel aquaporin 4 (AQP4) are found in the vast majority of patients with neuromyelitis optica spectrum disorders (NMOSD). In NMOSD, these autoantibodies bind to AQP4 on astrocytes [1, 2], and induce damage of these cells by antibody-dependent and complement-mediated (cellular) cytotoxicity. This leads to the formation of large astrocyte-destructive lesions in the central nervous system (CNS), followed by neuronal loss and secondary demyelination. Despite extensive research on NMOSD, the mechanisms behind the formation of pathogenic AQP4-specific antibodies remain elusive. Pathogenic AQP4-specific antibodies could arise as a consequence of impaired checkpoints of early B cell tolerance [3, 4]. However, pathogenic AQP4-specific antibodies could also form as consequence of paraneoplastic events ([5–8]), or in sequel to infections (e.g. tuberculosis, different herpes virus infections, Dengue virus infections [9, 10], hepatitis, or events summarized as “common cold” and “feverish infection” (for review see [5]). This suggests the action of viral or bacterial proteins mimicking antibody targets as first initiating events, as seen in patients with Guillain Barré Syndrome [11, 12] or some other antibody-driven diseases [13, 14]. Based on such observations we reasoned that one might be able to induce AQP4-reactive antibodies with mimotopes. Mimotopes are linear peptides which can fold themselves into short structural elements [15], and which mimic the target epitope of a given antibody so closely, that they are actually recognized by this antibody as well [16]. They can block the interactions between this antibody and its real target [17], and evoke similar responses when used as immunogens/vaccines [18], even if they do not share amino acid sequences with the recognized target. Most importantly, mimotopes can also mimic conformational epitopes [17, 19–22]. Therefore, they can be used in replacement of antigens which are difficult to isolate, or which lose their correct three-dimensional structure along with appropriately displayed conformational epitopes upon isolation [23].

We searched for mimotopes of the conformational AQP4 epitopes recognized by pathogenic AQP4-specific antibodies of NMOSD patients, and used them for the induction of AQP4-reactive antibodies in Lewis rats.

## Material and methods

### Animals

Lewis rats were used throughout this study. They were obtained from Charles River Wiga (Sulzfeld, Germany) and entered the experiments at an age of 8 weeks. The animals were housed in the Decentral Facilities of the Institute for Biomedical Research (Medical University Vienna) under standardized conditions.

### Patient samples

Plasmapheresates/serum samples from AQP4-antibody positive NMOSD patients were used for NMO-IgG preparations as described previously [24], and were adjusted to a concentration of 10 mg IgG/ml.

### Characterization of NMO-IgG preparations used for mimotope search

Testing their ability to bind to rat astrocytes. We first produced astrocyte-enriched cultures from neonatal rat brains following standard procedures [25]. Briefly, 0–24-h-old Lewis rats were killed and their brains dissected and transferred to RPMI 1640/10% fetal calf serum (FCS). The meninges were removed and the brains mechanically dissociated by gentle pipetting to obtain single cell suspensions which were cultured in poly-l-lysine-coated culture flasks with RPMI1650/10% FCS. After 5–7 days, the resulting cultures consisted of a monolayer of astrocytes and fibroblasts, with loosely adherent, ramified microglial cells and glial progenitor cells on top. These cultures were then agitated for 12–15 h (180 rpm, 37 °C) to detach the loosely adherent cells, which were then discarded. The firmly adherent cells (mostly astrocytes and fibroblasts) were rinsed with PBS, trypsinized, re-seeded onto poly-l-lysine-coated cover slips, and cultured for an additional 1–2 days in RPMI 1640/10% FCS. About 75% of all cells in these cultures were astrocytes. The cells were washed three times with RPMI 1640 before the cover slips were transferred to 100 μl drops of the NMO-IgG preparations (1:1000 in RPMI 1640/10% FCS), each in one well of a 12-well plate (Greiner Bio-One, Kremsmünster, Austria) and incubated for 30 min at 4 °C in a humidity chamber on a shaking platform (3 rpm). All following reactions were made under protection from light. After washing three times with ice-cold RPMI, donkey-anti-human-Cy3 antibodies (Jackson ImmunoResearch, West Grove, PA, USA, 1:100 in mixed glia medium) was applied for 45 min at 4 °C in a humidity chamber in the dark. Cells were washed three times with 1x PBS and then fixed with 4% PFA for 15 min at room temperature (RT). After another three times of washing with 1x PBS, the cells were permeabilized with 0.1% Triton-X-100 (Sigma-Aldrich) in RPMI/10% FCS for 5 min at RT and again washed with 1x PBS. Then, the cells were incubated with primary antibodies (rabbit-anti-AQP4 and goat-anti-GFAP, 1:100 in PBS/10% DAKO Real™ Antibody Diluent (Agilent Technologies Inc., Santa Carla, CA, USA) overnight at 4 °C in a humidity chamber on a shaking platform (3 rpm). Afterwards, the cells were washed three times with 1x PBS and secondary antibodies (donkey-anti-rabbit-Cy 2, 1:150, donkey-anti-goat-Cy5, 1:100 in DAKO Wash buffer (Agilent Technologies Inc.)/10% FCS) were applied for 1 h at RT. Having washed the cells three times with 1x PBS, 4′, 6-diamidino-2-phenylindole (DAPI) (Carl Roth GmbH und Co KG; 1:10000 in double-distilled water (ddH_2_O)) was applied for 5 min at RT. After washing with 1x PBS and with ddH_2_O, cover slips were mounted in one drop of Gallate/Geltol (Sigma-Aldrich) on glass slides and stored at 4 °C under protection from light. The stainings were analyzed using a Leica TCS SP5 setup (Leica Microsystems, CMS-GmbH, Germany). Cy2 signals were detected with an argon laser (488 nm excitation), detection of Cy3 was carried out using a DPSS561 laser and DAPI staining was detected with a 405 Diode laser. Images were edited and auto-adapted for contrast and brightness with ImageJ.

Testing their ability to initiate the formation of astrocyte-destructive lesions. This was done using standard procedures of our laboratory [24, 26]. Briefly, T cell lines against myelin basic protein (MBP, from guinea pig, Sigma) were intraperitoneally injected to induce experimental autoimmune encephalomyelitis (EAE). When the animals started to lose weight as first clinical symptom of EAE, they were intra-peritoneally injected with 1 ml phosphate buffered saline (PBS) containing either 10 mg NMO-IgG or 10 mg normal human IgG (Subcuvia^R^). 24 h later, the animals were sacrificed with CO_2_ and perfused with 4% phosphate buffered paraformaldehyde (PFA). Brains and spinal cords were dissected, immersed for another 18 h in PFA, and embedded in paraffin.

2–4 μm thick sections were cut on a microtome. The sections were dewaxed in xylol for 30 min, transferred to 96% ethanol, and incubated in 0.2% hydrogen peroxide for 30 min to block endogenous peroxidase. Then, the sections were rehydrated through a descending ethanol series (96, 70, 50%), rinsed in distilled water, and subjected to antigen retrieval by heating them for 60 min in 10 mM EDTA pH 8.5 in a conventional household steamer. Subsequently, the sections were rinsed in 0.1 M PBS or Tris-buffered saline (TBS) for 60 min, and exposed to 10% fetal calf serum (FCS) in 1 x DAKO Wash Buffer in PBS for 20 min at room temperature to reduce non-specific background. Then, immunohistochemical stainings were done essentially as described [24], using polyclonal rabbit anti-rat AQP4 (1:250, Sigma-Aldrich, Vienna, Austria) and the polyclonal rabbit anti-cow glial fibrillary acidic protein (GFAP, cross-reactive with rat; 1:3000; DakoCytomation), Immunohistochemistry was completed by incubation with biotinylated donkey anti-rabbit antibodies (1:2000, Jackson ImmunoResearch) followed by exposure to avidin-peroxidase complex (1:100 in DB/FCS; Sigma). Labeling was visualized with 3,3′ diaminobenzidine-tetra-hydrochloride (DAB, Sigma) containing 0,01% hydrogen peroxide. All sections were counterstained with Meyer’s hematoxylin, dehydrated and mounted in Eukitt© (Merck, Darmstadt, Germany).

### Mimotope search

The NMO-IgG preparations I-IV were used for mimotope searches. Details about NMO-IgG titers, age, gender, clinical disease, co-existing autoimmunity, and treatments at the time of sampling are summarized in Table 1.

**Table 1:** Characteristics of the NMO-IgG preparations used for mimotope search

NMO-IgG	Disease history of patient	characteristics	titer
**I**	38 year-old female patient; diagnosed with SLE in 1999, very mild NMO (transverse myelitis) in 2010; samples from 2013; patient has antibodies against AQP4 of both IgM and IgG1 isotypes. Treated with MTP and MMF at time of sampling	ANA+ (nuclei+, mitosis+, nucleoli-)	1:10240
**II**	68 year-old female patient with 5 years disease duration. Optic neuritis and thoracic myelitis (march 2010). Weakness of right leg and somnolence with diffuse brain lesions on left hemisphere and diffuse lesions in corpus callosum (july 2011, treated with steroids). Weakness of left leg with thoracic cord relapse (Th2~5), left vision disabled (september 2011).	Negative for ANA, SS-A/Ro, and SS-B/La [27]	1:8 × 10^6^
**III**	51 year-old female patient with optic neuritis; no episode of myelitis; no brain lesions (june 2013). Thymoma post-op (40y)	Negative for antibodies against SS-A, SS-B, Cardiolipin, MPO/C-ANCA, thyroglobulin, ribosomal P-protein, Scl-70, cyclic citrullinated peptide; ANA+, anti-dsDNA+,	1:640
**IV**	Female, African-Caribbean; no other morbidities. Disease onset at the age of 37 years-old when presented with LETM. Had other 5 attacks of either ON or LETM. Was left with unilateral blindness and mild lower limb deficits. Blood sample was taken when patient was stable, on Rituximab.	Negative for ANA	1:81920

**Fig. 1:**
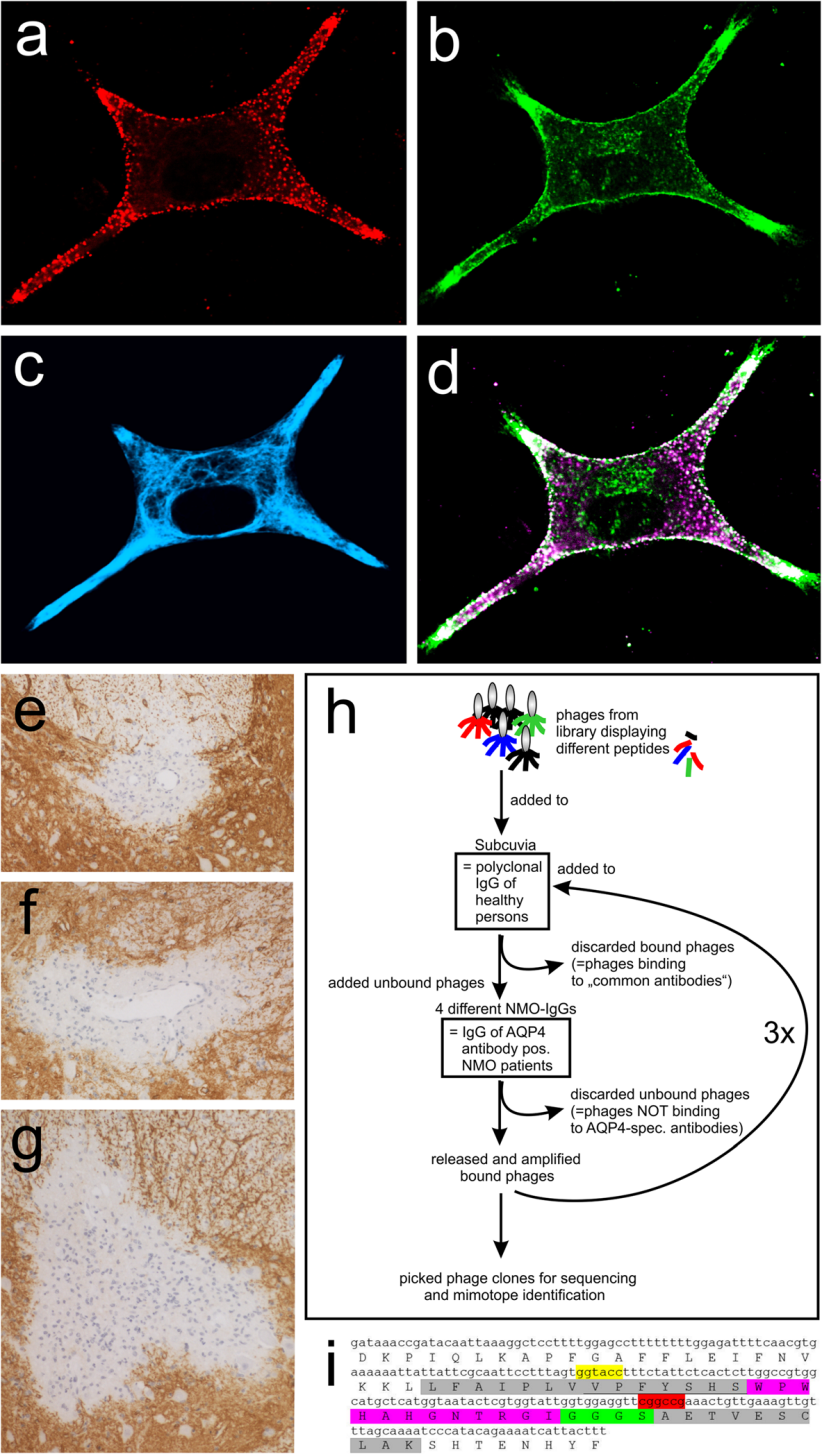
Characterization of NMO-IgGs used for mimotope search. **a**-**d** Immunofluorescence staining of Lewis rat astrocytes and analysis by confocal microscopy. The NMO-IgG preparation IV containing pathogenic AQP4-reactive antibodies recognizing conformational epitopes on the surface of astrocytes (**a**, red), a commercial AQP4-reactive antibody recognizing intracellular AQP4 epitopes (**b**, green) and an antibody directed against GFAP (**c**, blue) were used for stainings. Stainings against surface and intracellular AQP4 epitopes were merged to prove that IV contains AQP4-reactive antibodies reacting with rat AQP4 (**d**, white). **e**-**f** Formation of astrocyte-destructive lesions in experimental NMO. Shown here are spinal cords of Lewis rats injected with myelin basic protein-specific T cells and the NMO-IgG preparations IV (**e**), III (**f**) and I (**g**). Sections were stained with antibodies against AQP4 to show astrocytes (brown) and counterstained with hematoxylin to show nuclei (blue). **h** For each NMO-IgG preparation used, the phage display peptide library Ph.D.-12 was subjected to three rounds of negative selection on human control-IgG (Subcuvia) to deplete phages binding to “common antibodies”, and of positive selection on NMO-IgG to enrich for phages binding to the AQP4-reactive antibodies contained within the NMO-IgG preparation. At the end of these selections, bound phages were released, amplified, and sequenced for the identification of the mimotopes. **i** Example of sequencing results for mimotope IV-04. The DNA sequence represents the genomic (+) ssDNA in 5´➔ 3´ direction. Underneath you see the corresponding amino acid sequence (capital letters). Mimotope flanking regions are shown in gray, restriction enzyme recognitions sites in yellow and red, and the mimotope sequence in magenta

The NMO-IgG preparations were used together with the phage display peptide library Ph.D.-12 (New England Biolabs (NEB), Ipswich, MA). This library contains phages displaying 5 copies of a single randomized amino acid 12-mer fused via a glycin-glycin-glycin-serin (GGGS) linker to each of the five copies of the outer minor phage coat protein (pIII) of the M13 phage M13KE (information from the manufacturer). The library was essentially used according to NEB’s instructions, using three rounds of biopanning by negative selection on human IgG (Subcuvia™, Baxter, Vienna, Austria) followed by positive selection on NMO-IgG (Fig. 1). A detailed description is found in the supplementary information (S1). From aliquots of 98 phage clones binding to NMO-IgG after three rounds of negative/positive selection, and of 8 phage clones randomly picked prior to the selections (for usage as negative controls) DNA was isolated and sent for sequencing (VBC-Biotech Service GmbH, Vienna, Austria). Afterwards, the resulting DNA sequences were translated to amino acid sequences using the ExPASy translate tool (web.expasy.org/translate) to obtain information about the 12 amino acid-long peptides displayed by the analyzed phages. Details about DNA isolation and peptide quality controls are found in the supplementary information S2, S3.

The 12-mer amino acids displayed by the best NMO-IgG binders were then synthesized with a C-terminal GGGS extension by Centic Biotec (Heidelberg, Germany) or JPT Peptide Technologies GmbH (Berlin, Germany), with or without N-terminal acetylation and C-terminal amidation. We did not observe any differences between the different peptides containing these additional modifications or not, and used both of them without further discrimination for target enrichment ELISA.

To differentiate mimotopes - i.e. peptides binding to the antigen recognition site of AQP4-specific antibodies in the NMO-IgG preparation and thus mimicking the conformational AQP4 epitope - from peptides binding elsewhere onto the antibodies, antibody blocking assays were made (see supplementary information S4 for details).

### Mapping conformational epitopes

The peptides were mapped with Pepsurf (http://pepitope.tau.ac.il/; [28]) onto the 3D structure of human AQP4 (protein data base (pdb) accession number 3GD8) to identify potential epitope sites on AQP4. The BLOSUM62 matrix was used with a gap penalty of − 0.5. Algorithm parameters concerning library type and stop codon modification were adjusted for the Ph.D.™-12 (Phage Display Peptide-12) Library (library type: NNK; stopcodon modification: UAG replaced by glutamine). Only solvent exposed residues were considered to be parts of an epitope, available for antibody binding. A residue is regarded as exposed by Pepsurf if the solvent accessible surface area in the 3D structure is > 5% of its maximal accessible surface area. This calculation results in an estimated accessibility of the residues. The final alignment represents the best possible path in a defined surface graph. The probability obtaining the same alignment with a random sequence is given by the corresponding *p*-value.

### Antibody induction in Lewis rats

Immunization protocol 1. Animals (8 rats/peptide) were subcutaneously immunized with mimotope-KLH at 4 different sites along the flanks with a total amount of 200 μl of a suspension containing 20 μl Alum (Alhydrogel, InvivoGen, San Diego, USA), 20 μl 10xPBS, and 160 μl peptide (see Table 2; stock 5 mg/ml, dissolved in 30% DMSO), and were boosted bi-weekly with the same suspension, starting 2 weeks after the primary immunization. At the time when the animals were boosted, serum was taken to test for the presence of anti-rat AQP4-antibodies.

**Table 2:** Amino acid sequences of mimotopes, peptides, and their derivatives for immunization

**Mimotope ID**	**Mimotope:** **Amino acid sequences including GGGS linker**	**Mimotope-KLH:** **Mimotope-keyhole limpet hemocyanin fusions for immunization protocol 1**	**Mimotope-AQP4**_**268–285**_: **Mimotopes fused with AQP4-reactive T cell epitope used for immunization protocol 2**
I-04	WRYHVHPTPFKSGGGS	WRYHVHPTPFKSGGGSC- keyhole limpet hemocyanin	WRYHVHPTPFKSKAAQQTKGSYMEVEDNRS
I-13	GPFHFLHHHWSQGGGS	GPFHFLHHHWSQGGGSC- keyhole limpet hemocyanin	GPFHFLHHHWSQKAAQQTKGSYMEVEDNRS
I-18	WSSHAHRHNHFRGGGS	–	
IV-04	WPWHAHGNTRGIGGGS	WPWHAHGNTRGIGGGSC- keyhole limpet hemocyanin	WPWHAHGNTRGIKAAQQTKGSYMEVEDNRS
IV-27	IQYAPGGSYSVIGGGS	–	
IV-38	VKGHWHHLNHANGGGS	–	VKGHWHHLNHANKAAQQTKGSYMEVEDNRS
II-01	FPFWHRTHAWDRGGGS	–	
III-01	WSWKHHHPIMPRGGGS	WSWKHHHPIMPRGGGSC-keyhole limpet hemocyanin	WSWKHHHPIMPRKAAQQTKGSYMEVEDNRS
		“mix”: I-04 (WRYHVHPTPFKSGGGSC- keyhole limpet hemocyanin) + I-13 (GPFHFLHHHWSQGGGSC- keyhole limpet hemocyanin) + IV-04 (WPWHAHGNTRGIGGGSC- keyhole limpet hemocyanin) + III-01 (WSWKHHHPIMPRGGGSC-keyhole limpet hemocyanin)	–
Neg.controls:		–	–
II-17	HFWGHHRTTSKVGGGS	–	–
random peptide L5	SPRAISSYPLNEGGGS	–	–
random peptide L8	FPTDSLRGDVGMGGGS	–	–

There are no matching linear sequences of the mimotopes to human AQP4 loops A (TINWGGTEKPLPVD), C (TPPSVVGGLGVTMVHGNLT) or E (NWENHW)

Loop sequences were taken from [29]

The animals were killed by CO_2_ inhalation, and brains, spinal cords and kidneys prepared for immunohistochemical analysis as outlined above.

Immunization protocol 2. Animals were subcutaneously immunized at 4 different sites along the flanks with a total amount of 200 μl of a 1:1 mixture of mimotope-AQP4_268–285_ (Table 2, stock 1 mg/ml) in Freund’s incomplete adjuvans supplemented with 4 mg/ml *Mycobacterium tuberculosis* H37Ra (Complete Freund’s adjuvans, CFA). 5–6 weeks later, the animals were boosted with the same antigen/adjuvans mixtures (total amount 200 μl, distributed over 4 sites along the flanks), and killed 2 weeks later by CO_2_ inhalation. Blood was taken for serum analysis. Brains, spinal cords, and kidneys were dissected, post-fixed in 4% paraformaldehyde in phosphate-buffered saline (4%PFA) for additional 18–24 h and embedded in paraffin for histological analysis.

Both immunization protocols did not induce any clinical symptoms in the experimental animals.

### Titer determinations

Serum was used to determine the titers of AQP4-reactive antibodies in cell-based assays as described, using HEK293 cells transfected with rat or human AQP4 M23 as targets [24, 30].

### Identification of IgG subclasses of AQP4-reactive antibodies in live cell-based assays

IgG subclasses of mimotope-immunized rat sera were investigated using a live cell-based-assay with HEK293 cells expressing rat-AQP4 fused to EmGFP [31]. Sera from four different animals were studied. The mouse monoclonal AQP4 antibody E5415A [32] was used as a positive control. Sera taken from a mimotope-immunized rat with an antibody titer of zero and from a normal healthy control rat served as negative controls.

HEK293 cells expressing rat AQP4-EmGFP were blocked with goat-IgG (4 μg/ml; Sigma-Aldrich) and subsequently incubated with rat sera diluted 1:20 and 1:40 in 10%FCS/PBS (FCS, Gibco; PBS, Sigma-Aldrich) or with the mouse monoclonal AQP4 antibody E5415A (5 μg/ml in 10%FCS/PBS) for 1 h at 4 °C. After washing three times with 10%FCS/PBS, IgG subclasses were determined by incubating with mouse anti-rat IgG1, IgG2a, IgG2b and IgG2c antibodies (1:200 in 10%FCS/PBS; BioLegend) for 30 min at 4 °C. Following a washing step cells were incubated with Alexa Fluor 594 labelled goat anti-mouse IgG (1:500 in 10%FCS/PBS; Invitrogen) at room temperature for 30 min. Dead cells were excluded by DAPI staining (Sigma-Aldrich). Data were analyzed by two investigators (ML and KS).

### Studying the ability of mimotope-induced AQP4-reactive antibodies to induce complement-dependent cytotoxicity

A live cell-based assay with HEK293 cells expressing rat-AQP4 fused to EmGFP was used to analyze antibody-mediated complement activation [31]. Five different rat serum samples were studied together with 7 positive controls (mouse monoclonal AQP4 antibody E5415A, 2 rat sera containing the E5415A antibody, 3 human AQP4-Ab positive NMOSD serum samples and 1 human NMOSD plasmapheresis sample) and 3 negative controls (rat serum, mouse IgG and serum from a mimotope immunized rat with an antibody titer of zero).

Briefly, serum samples and an aliquot of rat complement serum (Dunn Labortechnik, Asbach, Germany) were heat inactivated for 1 h at 56 °C. HEK293 cells expressing rat-AQP4EmGFP were blocked with goat IgG (Sigma-Aldrich, 4 μg/ml in X-VIVO (Lonza)), washed three times with X-VIVO and subsequently incubated with serum samples (diluted 1:10 in X-VIVO), the monoclonal AQP4 antibody E5415A (20 μg/ml in X-VIVO) or mouse IgG (Sigma-Aldrich, 20 μg/ml in X-VIVO) and with 20% active or 20% heat-inactivated rat complement for 90 min at 37 °C. For detection of the terminal complement complex (TCC) cells were washed three times with X-VIVO and incubated with the murine monoclonal anti-rat C5b-9 antibody (eubio, Vienna, Austria, 2 μg/ml in X-VIVO) for 1 h at 4 °C. Next, after washing three times with X-VIVO, cells were incubated with Alexa Fluor 594 labelled goat anti-mouse (Invitrogen, diluted 1:500 in X-VIVO) for 30 min at room temperature. Following a washing step with PBS/10%FCS, dead cells were visualized by DAPI staining (Sigma-Aldrich).

### Search for homologous sequences

Homology searches were made with the basic local alignment search tool (BLAST) (http://blast.ncbi.nlm.nih.gov/Blast.cgi), using the dodecamer peptide sequences as input for the search algorithm blastp (protein-protein BLAST) and the program BLASTP 2.2.0+ to probe the non-redundant database (including all non-redundant GenBank (gb) coding sequences (CDS) translations, the protein data bank (PDB), SwissProt, the protein information resource (PIR) database, and the protein research foundation (PRF) database excluding environmental samples from whole genome shotgun (WGS) projects). Searches were either done unlimited (for the detection of mimotope-related sequences in bacteria/fungi/parasites), or limited to RNA viruses (taxid: 2559587), *Mycobacterium tuberculosis* typus humanus (taxid: 1773), or Helicobacter pylori (taxid: 2010). Only the top Blast Hits on 100 subject sequences were analyzed for each search.

### Statistical analysis

Statistics were calculated with GraphPad Prism 7.00.

## Results

### The NMO-IgGs used for mimotope search react with AQP4 on rat astrocytes and initiate the formation of astrocyte-destructive lesions in a Lewis rat model of NMOSD

All the NMO-IgGs used for mimotope search derived from patient sera or plasmapheresates which were found positive for pathogenic antibodies against human AQP4 in standard live cell-based diagnostic assays [33] (Table 1). These antibodies also reacted with AQP4 of rat astrocytes (Fig. 1, Supplementary information S5). To test whether the NMO-IgG preparations I, III, and IV are also able to initiate astrocyte destructive lesions in our rat model of experimental NMOSD [24], we injected these NMO-IgGs into Lewis rats after opening the blood-brain barrier of these animals with MBP-specific T cells. All three NMO-IgGs initiated the formation of typical perivascular lesions with loss of AQP4 (Fig. 1) and of GFAP reactivity (data not shown) indicative of astrocyte-destruction. For the NMO-IgG preparation II, these features have already been confirmed before [27]. Hence, the NMO-IgG preparations used for mimotope search were proven pathogenic in Lewis rats.

### Identification of mimotopes representing extracellular epitopes of human AQP4

**Fig. 2:**
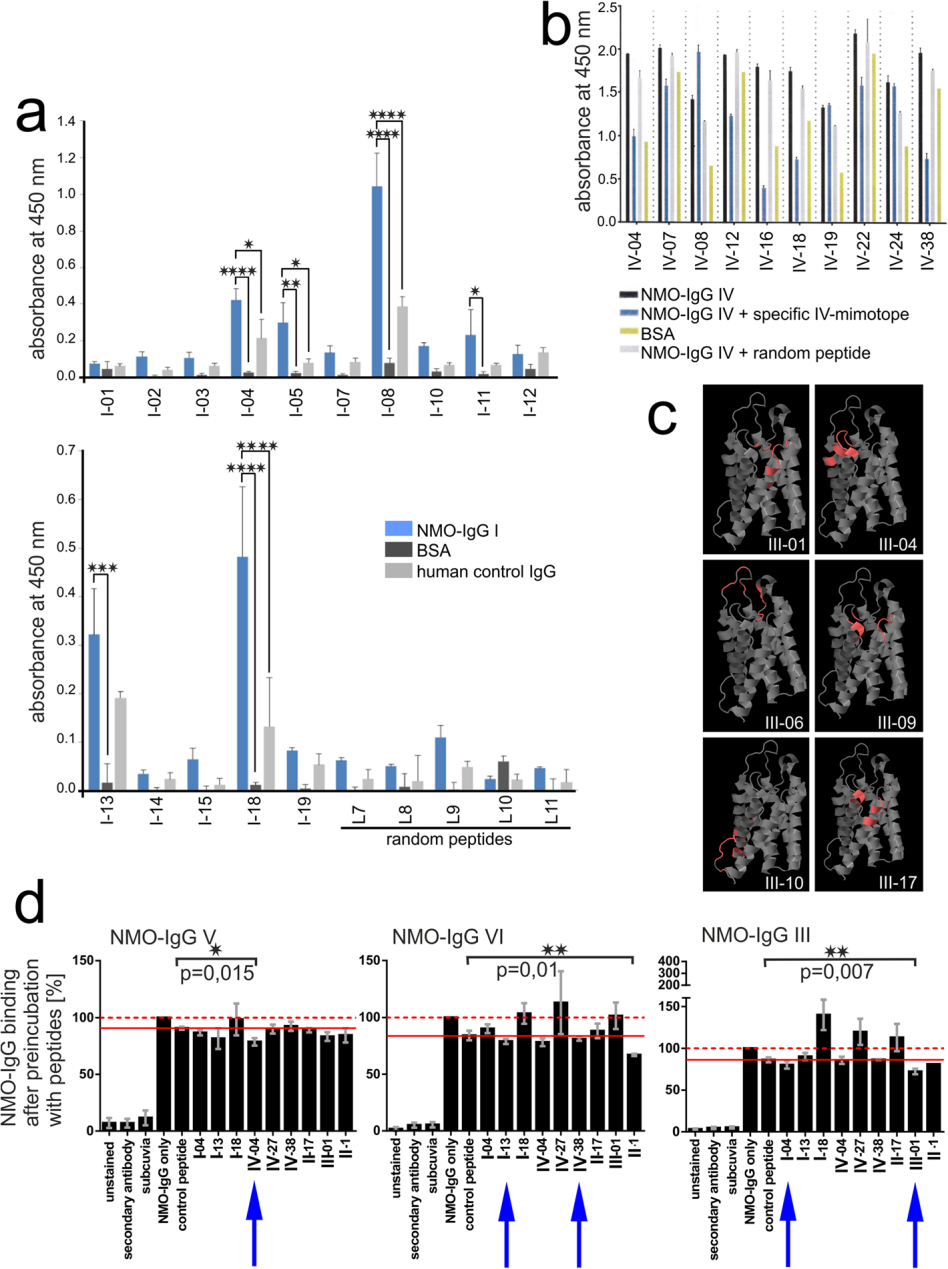
Characterization of phage clones, phage-displayed peptides, and mimotopes. **a**-**c** For different NMO-IgG preparations, different experimental approaches were made to narrow down the number of phage-displayed peptides for further studies. **a** ELISA with single phage clones to evaluate interactions with the NMO-IgG preparation I. Single phage clones after 3 rounds of negative/positive selection (I-01 - > I-19) and 5 randomly picked phage clones without preceeding selection (L7 - > L11) were tested for their ability to react with the NMO-IgG preparation I, BSA, or control-IgG (Subcuvia). Each sample contained 10^8^ phages. Data represent three different experiments and are shown as mean + SEM (*p < 0.05; **p < 0.01; ***p < 0.001; ****p < 0.0001, detected with one-way ANOVA followed by Sidak’s multiple comparisons test). **b** ELISA to verify that peptides, but not phage particles bind to the NMO-IgG preparation IV. IV was pre-incubated with specific, random, or no synthesized peptides prior to the incubation with peptide-displaying phages. Bound phages were then detected with horse radish peroxidase-conjugated anti-M13 antibodies and TMB substrate, and the absorbance was measured at 450 nm in an ELISA reader. Data represent triplicates of one experiment and are shown as mean + SEM. **c** Phage-displayed peptides identified with the NMO-IgG preparation III were mapped onto the 3D structure of human AQP4 (protein data base (pdb) accession number 3GD8) using PepSurf. Extracellular loops are displayed on top of the structure, intracellular parts of the molecule on the bottom of the 3D structure. The final alignment represents the best possible path in a defined surface graph. The probability obtaining the same alignment with a random sequence is given by the corresponding *p*-value. III-01 (*P*-value: 0.00017), III-04 (P-value: 0.00106), III-06 (P-value: 0.00007), and III-09 (P-value: 0.00028) mapped at least partially to the extracellular loops of AQP4 (red), while III-10 (P-value: 0.00008) and III-17 (P-value: 0.00047) mapped to intracellular or helical structures, respectively. **d** Selected peptides were tested for their ability to interfere with the binding of AQP4-reactive antibodies of NMO-IgG preparations to AQP4. Flow cytometry of AQP4 M23-transfected HEK293A cells reacting with NMO-IgG preparations V, VI, and III pre-incubated with the indicated peptides. Pre-incubation without peptide, or with a random peptide (SPRAISSYPLNEGGGS) served as negative controls. The data shown here are the mean values (+/−SEM) of the percentage of NMO-IgG binding after pre-incubation with peptides, obtained from 4 (V and III) or 5 (VI) independently performed experiments. Please note that the binding of the NMO-IgG preparations pre-incubated without peptide or with random peptides slightly differed from each other. Therefore, we referred to the binding of NMO-IgG without peptide as 100% (dashed red line) and always also show the percentage of binding of NMO-IgG pre-incubated with random peptide (solid red line). The lower one of these two different values was used as reference for the percentage of blocking achieved with the different peptides. Statistics was calculated using one-tailed, Welch-corrected t-tests. Blue arrows indicate mimotopes used for immunization

We used the phage display library Ph.D.-12 and 3 alternating rounds of negative selection on control human IgG (Subcuvia™) to deplete phages with peptides binding to irrelevant antibodies or serum proteins, and of positive selection on single NMO-IgGs to enrich phages displaying peptides binding to the AQP4-reactive antibodies (Fig. 1). At the end of these selections, single phage clones were sequenced to gain information about the amino acid sequences of the phage-displayed 12-mer peptides (Fig. 1). For further studies, peptides were first pre-selected based on high binding rates to NMO-IgG in ELISA (Fig. 2), on their interference with phage-binding to NMO-IgG (Fig. 2), or on their mapping to extracellular loops of human AQP4 (pdb 3GD8) (Fig. 2), before we tested whether the selected peptides represent true mimotopes able to compete with conformational AQP4 epitopes on human AQP4-transfected HEK293 cells for NMO-IgG binding (Fig. 2). None of the tested peptides was able to completely interfere with NMO-IgG binding, and those which were able to interfere at all did so very poorly (Fig. 2, and supplementary information S4). This might be due in part to the polyclonal nature of NMO-IgGs [29, 34], and could also result from higher affinities of the NMO-IgGs for the natural ligand than for the tested peptides. We did not further address this point, but concentrated on peptides which significantly interfered with the binding of AQP4-reactive antibodies from at least 1/15 different NMO-IgG preparations (IV-04, II-1, and III-01, Fig. 2 and Table 2), and on those which showed some interference with these NMO-IgGs in the same tests, even if this did not reach significance (I-04, I-13, and IV-38, Fig. 2 and Table 2). These peptides had a completely different amino acid sequence than the three extracellular loops of AQP4 (Table 2), and were subsequently termed mimotopes. 5 of them (IV-04, III-01, I-04, I-13, and IV-38) were used for the immunization of Lewis rats (Table 2).

### Immunization of Lewis rats with AQP4 mimotopes induces the production of AQP4-reactive antibodies which recognize conformational epitopes on the surface of HEK293 cells transfected with rat AQP4, and cross-react with human AQP4

The animals were immunized with two different protocols. First, we immunized Lewis rats with mimotope-KLH/alum (Table 2), and boosted the animals 4x with the same immunogen. This approach let to very low AQP4-reactive antibody titers in very few animals: Only 2/32 animals reached antibody titers > 20: One animal had titers of 40/40/n.d./80 measured 2 weeks apart, the other had titers of 20/80/40/20 in the same time period. All others were essentially negative after the 4th boost.

**Fig. 3:**
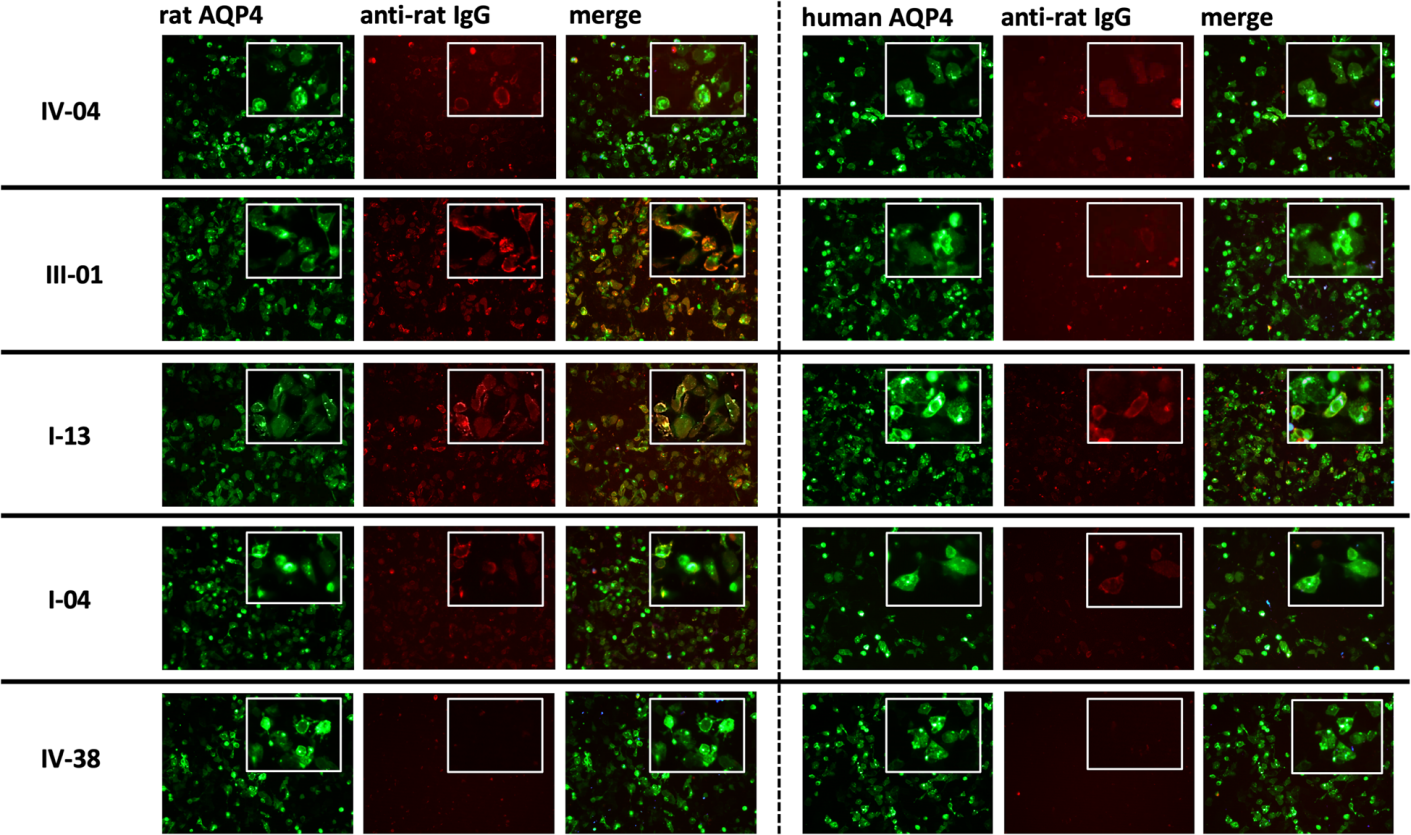
Detection of AQP4-specific antibodies in sera of Lewis rats immunized with mimotopes. Life-cell immunofluorescence staining against the surface antigens rat AQP4-EmGFP and human AQP4-EmGFP was made. Antigens were transiently transfected into HEK293 cells (green) and sera of Lewis rats immunized with mimotopes IV-04, III-01, I-13, I-04, or IV-38 were added. Bound antibodies were then detected with anti-rat-IgG (red). Merge of green and red signals reveals surface staining of transfected cells in yellow if antibody signals were strong. Additional images at 40x magnification were added for details (see inlay). For titer values see Table 3

For the second protocol, we immunized the animals with mimotopes fused to AQP4_268–285_ which contains two overlapping, highly encephalitogenic T cell epitopes of the AQP4-response in Lewis rats [35]. Most importantly, the sequence AQP4_268–285_ does not contribute to the extracellular loops A, C, and E of AQP4 [29, 36]. We used CFA as adjuvant (Table 2), and boosted the animals 5–6 weeks later with the same immunogen. 2 weeks after boosting, at the time of sacrifice, all animals appeared healthy, and did not display any clinical signs of NMOSD. Histological evaluation revealed that none of the immunized animals had any inflammatory CNS lesion or CNS lesion with AQP4 loss typical for NMOSD (data not shown). We then studied the sera taken at the time of sacrifice, and analyzed them with the same live cell-based assays used for the detection of pathogenic AQP4-reactive antibodies in NMOSD patients [33]. We found AQP4-reactive antibodies above threshold (titer > 20) in 3/8 rats immunized with III-01-AQP4_268–285_/CFA, in 3/5 animals immunized with I-13-AQP4_268–285_/CFA, in 4/5 animals immunized with I-04-AQP4_268–285_/CFA, in 3/5 animals immunized with IV-04-AQP4_268–285_/CFA, and in 0/4 animals immunized with IV-38-AQP4_268–285_/CFA (Table 3). The antibodies recognized conformational AQP4 epitopes on the surface of HEK293 cells transfected with human AQP4 M23 (Table 3, Fig. 3, range of titers 40–160), and were also reacting with HEK293 cells transfected with rat AQP4 (Table 3, Fig. 3, range of titers 40–320). With serum from the I-04-AQP4_268–285_/CFA immunization series (anti-rat AQP4 titer 320), we could further confirm binding of the induced antibodies to rat astrocytes (Supplementary information S6). Hence, the AQP4-reactive antibodies produced by immunization with mimotope-AQP4_268–285_/CFA showed cross-reactivity between rat and human AQP4, as is typical for pathogenic AQP4-reactive antibodies found in NMOSD patients [24, 34].

**Table 3:** Antibody titers obtained with immunization protocol 2

**Mimotope** **(*n*** **= number of rats)**	**Titer** **Anti-rat AQP4**	**Titer** **Anti-human AQP4**	Considered antibody-positive according to cut-off criteria***
**III-01 (*n*** **= 8)**	**20**	**0**	
**∆**	**0**	**20**	
	**10**	**0**	
	**80**	**20**	***
	**0**	**0**	
	**40**	**20**	***
	**0**	**0**	
	**160**	**40**	***
**IV-38 (*n*** **= 4)**	**5**	**10**	
	**0**	**0**	
	**0**	**0**	
	**0**	**0**	
**I-13 (*n*** **= 5)**	**0**	**0**	
	**320**	**80**	***
	**160**	**0**	***
	**40**	**10**	***
	**0**	**0**	
***I-04 (n = 5)***	**40**	**10**	***
	**20**	**20**	
	**320**	**160**	***
	**40**	**40**	***
**∆**	**320**	**80**	***
***IV-04 (n = 5)***	**80**	**10**	***
	**0**	**0**	
	**160**	**0**	***
	**160**	**40**	***
	**10**	**0**	

***Titers considered positive when single values for anti-rat AQP4 or anti-human AQP4 > 20

∆ Serum used for staining of rat astrocytes (Supplementary information S6)

### Sera of mimotope-AQP4_268–285_-immunized Lewis rats contain AQP4-reactive antibodies with high and low complement-fixing isotypes

**Fig. 4:**
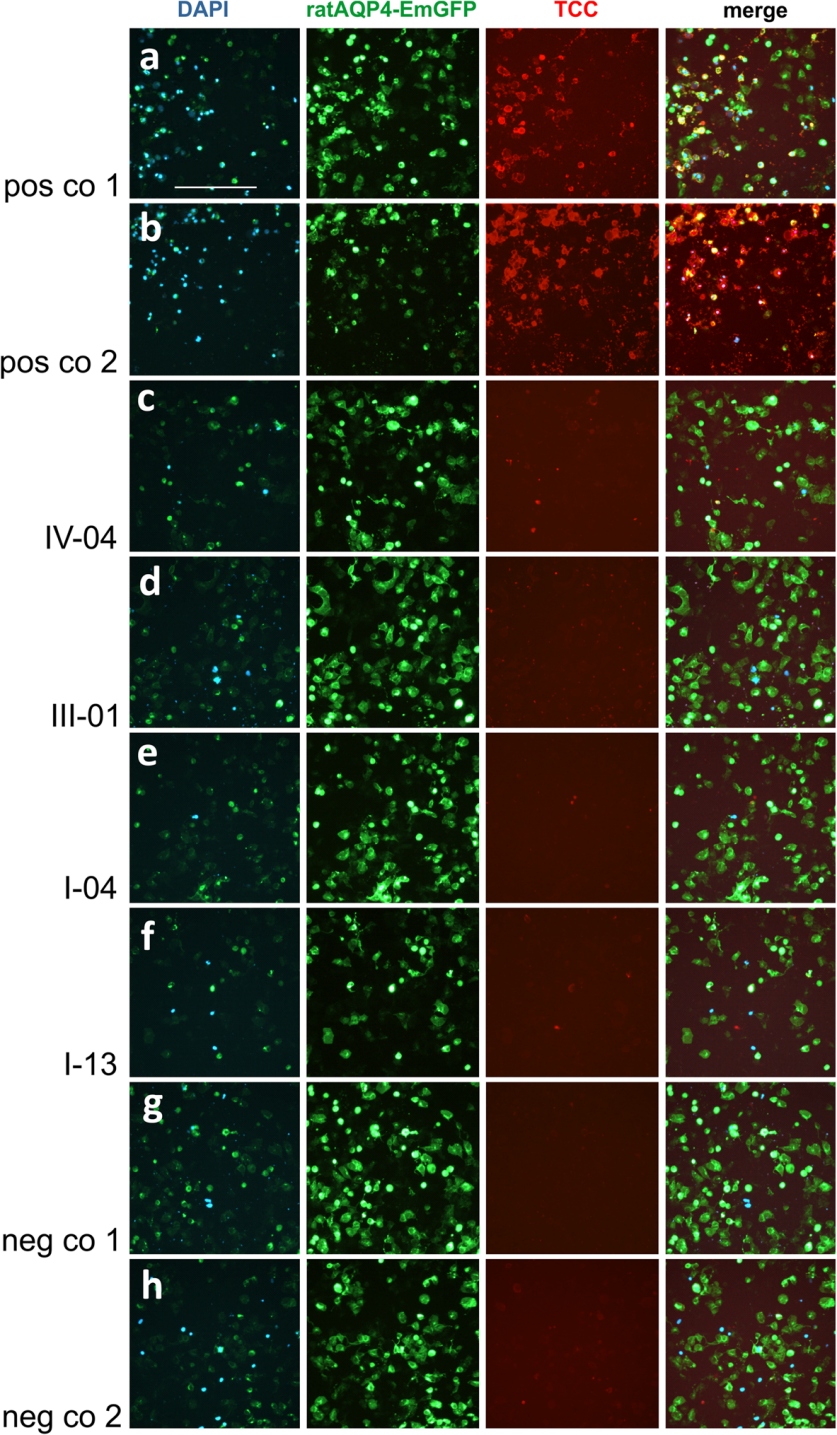
Antibody mediated complement activation of serum samples from rats immunized with AQP4 mimotopes. **a**: Serum AQP4 antibodies from an AQP4 antibody positive NMOSD patient (**a**) and the mouse monoclonal E5415A AQP4 antibody were able to activate the complement cascade in the presence of active rat complement on rat-AQP4-EmGFP (green) expressing HEK293 cells resulting in TCC deposition (red) and cell death (DAPI, blue). **a** and **b** are positive controls (pos co 1 and 2). **c**-**f**: Sera from rats immunized with mimotope-AQP4_268–285_/CFA (mimotopes were IV-04 inducing rat AQP4-spec antibodies in a titer of 1:160, III-01 inducing rat AQP4-spec antibodies in a titer of 1:160, I-04 inducing rat AQP4-spec antibodies in a titer of 1:320, and I-13 inducing rat AQP4-spec antibodies in a titer of 1:320) were not able to activate the complement cascade as shown by fewer DAPI (blue) positive cells and no TCC (red) deposition. **g**: Serum from a III-01-AQP4_268–285_/CFA immunized rat with an antibody titer of zero was used as a first negative control (neg co 1) and shows no antibody mediated complement activation. **h**: Same serum as in A in the presence of heat-inactivated rat complement (second negative control (neg co 2) does not activate the complement cascade and shows no TCC (red) formation. Scale bar = 100 μm

All serum samples from Lewis rats immunized with the different mimotope-AQP4_268–285_ (IV-04, III-01, I-04, or I-13) contained the AQP4-reactive antibodies as mixtures of low complement-binding isotypes IgG1 and high complement-activating isotypes, predominantly IgG2a and IgG2b [37] (Table 4, supplementary information S7). Despite the presence of AQP4-reactive antibodies with complement-fixing isotypes, the rat sera were unable to activate the complement cascade in vitro, when tested in live cell-based assays using HEK293 cells expressing rat-AQP4EmGFP as targets (Fig. 4). The most likely reason for this observation is that the high and low complement-fixing AQP4-reactive antibodies compete with each other at the binding sites and thus prevent that the critical threshold of complement-fixation is reached.

**Table 4:** Semiquantitative analysis of immunoglobulin G isotypes among AQP4-reactive antibodies raised in mimotope-AQP4_268–285_-immunized Lewis rats

	**IgG1**	**IgG2a**	**IgG2b**	**IgG2c**
**IV-04**	**+**	**+**	**+**	**+**
**III-01**	**+(+)**	**+++**	**+++**	**+(+)**
**I-04**	**+**	**++(+)**	**+(+)**	**+**
**I-13**	**++(+)**	**++**	**++**	**++**

+ to +++ indicate reactivity of immunoglobulin G isotype-specific antibodies to AQP4-reactive antibodies from mimotope-AQP4_268–285_-immunized Lewis rats bound to HEK293 cells expressing rat AQP4-EmGFP. The reactivity was rated as + (weak), ++ (moderate), and +++ (strong), respectively

Human AQP4 antibody-seropositive NMOSD samples (containing predominantly complement fixing AQP4-reactive antibodies of the IgG1 and IgG3 isotype [24]) and the monoclonal E5415A antibody (complement fixing murine IgG2a isotype) used as positive controls showed strong complement activation resulting in deposition of the terminal complement complex (TCC) on cell surfaces, and in higher cell mortality evidenced by increased DAPI staining (Fig. 4). In contrast, incubation with heat-inactivated rat complement or with rat serum and mouse IgG showed no TCC deposition and no activation of the complement cascade.

### Similarity of mimotopes to protein sequences of human pathogens

As shown above, our mimotopes efficiently mimic conformational epitopes of AQP4 for antigen recognition by AQP4-reactive antibodies. Since NMOSD patients may develop clinical disease in sequel to infections, and since molecular mimicry processes of infectious agents were already shown in the development of other autoimmune diseases [38–40], we next analyzed with BLAST whether our AQP4-mimotopes share linear amino acid sequences with proteins of human pathogens. Since antibodies typically recognize 4–12 amino acid-long epitopes, with most robust responses starting with 5 amino acids [41], we postulated that mimotope and pathogen-derived protein must share ≥5 amino acids in continuation plus ≥1 flanking shared amino acid or conservative amino acid substitution. We found 13 highly homologous amino acid sequences of human pathogens (Table 5). Some of these pathogens have been observed more frequently in NMOSD patients, like e.g. *H. pylori* [56, 64] or M. tuberculosis [57–60, 65], others were anecdotically reported in NMOSD patients, like e.g. Legionella [66]. We also identified pathogens which have never been described in the context of NMOSD, like *Pseudomonas aeruginosa*, Serratia spec, or *Enterobacter cloacae* complex spec.

**Table 5:** Sequence homology between different mimotopes and bacterial/fungal/parasitic proteins. All proteins are located intracellularly, but might be released as a consequence of immune-mediated damage

**Mimotope**	**# Mimotope sequence #** **Quality of match**^**a**^ **# Organismal protein sequence #**	**Protein name** ***Protein sequence ID*[Organism]**	**Possible disease** **association of bacteria/fungi/parasites or presence in human microbiome**
**III-01**	**5 HHHPIMPR 12** **HH + PIMPR** **183 HHQPIMPR 190**	DUF3306 domain-containing protein *WP_083797754.1* [Vibrio Metschnikovii].	Infections [42, 43]; Isolated cases of pneumonia [44]**.**
	**5 HHHPIMPR 12** **HHHPI + PR** **160 HHHPILPR 167**	N-succinylarginine dihydrolase *WP_058459851.1* [Fluoribacter bozemanae = Legionella bozemanae] Same alignment seen for Legionella anisa and Legionella longbeachae	Legionellosis [45, 46].
**I-04**	**3 YHVHPTPF 10** **Y + VHPTPF** **519 YQVHPTPF 526**	Hypothetical protein CORT_0F01650 *XP_003870521.1* [Candida orthopsilosis Co 90–125]	Rare infections [47]
	**3 YHVHPTP 9** **YHVHPTP** **560 YHVHPTP 566** **6 HPTPFK 11** **+PTPFK** **57 QPTPFK 62**	Vacuolar sorting-associated protein *EGC49414.1* [*Histoplasma capsulatum* H88];Same alignment seen forvacuolar protein sorting-associated protein 27 *EER41546.1* [Histoplasma capsulatum H143] Hypothetical protein *WP_080024432.1* [Helicobacter pylori]	Histoplasmosis [48] Gastritis, peptid ulcers [49]
**I-13**	**2 PFHFLHH 8** **PFHFLHH** **3450 PFHFLHH 3456** **7 HHHWSQ 12** **HHHWS+** **245 HHHWSH 250**	RAVE 1 carboxy-terminal protein *RQX74768.1* [Toxoplasma gondii] Chain A, Enoyl-CoA hydratase/isomerase family protein *4HC8_A* [*Mycobacterium tuberculosis*]	Toxoplasmosis [50] Tuberculosis [51]
**IV-04**	**1 WPWHAH-GNTRGI 12** **WPWHAH GN R I** **119 WPWHAHLGN-RVI 130** **1 WPWHAHGN 8** **W WHAHGN** **393 WAWHAHGN 400** **1 WPWHAHGNTR 10** **W WHAHGN R** **243 W-WHAHGNSR 251** **4 HAHGNTRGI 12** **HA GNTRG+** **252 HARGNTRGV 260** **5 AHGNTRGI 12** **A + GNTRG+** **253 AQGNTRGV 260** **1 WPWHAHGN 8** **W WHAHGN** **123 WRWHAHGN 130**	Glycosyltransferase family 2 protein *WP_040707114.1* [Nocardia takedensis]; Same alignment seen in [Nocardia pneumoniae] Gentisate 1,2-dioxygenase *VFT64169.1* [*Pseudomonas aeruginosa*] MFS transporter *WP_115184045.1* [Serratia multispecies, among them S. plymuthica, S. quinivorans, S. proteamaculans, S. liquefaciens, S. grimesii] Hop family adhesin BabA *CEI71366.1* [Helicobacter pylori] Hop family adhesin BabA *WP_000716277.1* [Helicobacter pylori] cupin domain-containing protein, partial *WP-129361314.1* [*Enterobacter cloacae* complex sp. 2DZ2F16B1]	Nocardiosis [52] Different types of infections [53] Different types of infections [54] Gastritis, peptid ulcers [49] Gastritis, peptid ulcers [49] Complex includes common nosocomial pathogens capable of producing a wide variety of infections [55]

Homology searches were made with the basic local alignment search tool (BLAST) and the dodecamer mimotope sequences as input. The combined results of the following searches were pooled for this table: unlimited search, search limited to *H. pylori* (taxid: 210), search limited to Mycobacterium tuberculosis typus humanus (taxid: 1773), and search limited to RNA viruses (taxid: 2559587). The limited searches were done due to possible associations of NMOSD with infections by H.pylori [56], M. tuberculosis [57–60], and Hepatitis viruses (RNA viruses] [61–63]. The top Blast Hits on 100 subject sequences were analyzed. As cut-off criteria, only continuous sequences ≥5 amino acids plus ≥1 flanking identical amino acid or positive match were considered

# represent the position of the amino acids in the mimotope or protein sequence

^a^amino acids found in both sequences are indicated by letters

+ = positive match (conservative substitution)

white space = match with zero/negative score

**-** = gap

The mimotope I-13 shared homology with a 6 amino acid long sequence of Chain A from the enoyl-CoA hydratase of M. tuberculosis (sequence 7 HHHWSQ 12 of I-13 compared to sequence 245 HHHWSH 250 of the M. tuberculosis protein, respectively; Table 5). Since the CFA used for mimotope-AQP4_268–285_ immunizations contained M. tuberculosis H37Ra, one could argue that the AQP4-reactive antibodies were induced by a protein of M. tuberculosis instead of the mimotopes. However, the following points argue against this possibility: Firstly, our mimotopes also produced AQP4-reactive antibodies in animals immunized with mimotope-KLH in alum. Secondly, sequences homologous to a M. tuberculosis protein were only seen for I-13, but not for III-01, I-04, or IV-04, which all gave rise to AQP4-reactive antibodies (Table 5), while M. tuberculosis proteins were also present in the immunizations with IV-38-AQP4_268–285_ in CFA, which did not lead to the production of AQP4-reactive antibodies (Table 3).

## Discussion

Anergy induction, activation-induced cell death or receptor editing are powerful tolerance mechanisms preventing the formation of autoantibodies in the organism [4]. However, autoantibodies may be induced when such checkpoints of early B cell tolerance are impaired [3], or when they are circumvented by proteins from infectious agents mimicking the antibody targets sufficiently well [11–13].

In the current study, we used a phage display library and searched for peptides mimicking the conformational epitopes of AQP4, i.e. for AQP4 mimotopes. We identified such peptides and show that they do not share homology in amino acid sequence to the extracellular loops of AQP4. At first glance, this seems surprising, but this might be due to the fact that we used polyclonal NMO-IgG preparations instead of monoclonal AQP4-specific antibodies for our search, and that different AQP4-specific antibodies may recognize different conformational epitopes formed by the extracellular loops of AQP4. Moreover, mimotopes do not have to share amino acid sequences with their corresponding epitope, but could just share a similar structure. This was seen for example for Rp10-L, a mimotope for the rituximab-specific CD20 epitope [67], or for H98, which induces anti-HER-2-specific antibodies, but does not share homology with HER-2 [68].

Here, we show that the identified peptides were able to compete with conformational AQP4 epitopes for the binding of patient-derived NMO-IgG. We then fused these mimotopes as surrogate B cell epitopes with immunodominant T cell epitopes of the anti-AQP4 response in Lewis rats and used these mimotope-AQP4_268–285_ constructs for the immunization of Lewis rats. These animals produced AQP4-reactive antibodies which bind to living AQP4-expressing cells in the same cell-based assays used for the identification of AQP4-antibody seropositive NMOSD patients [33]. Moreover, patient-derived and mimotope-AQP4_268–285_-induced AQP4-reactive antibodies recognize both human and rat AQP4, undergo T cell help-dependent class switch leading to the production of IgG, consist of different clones (see [34] for human AQP4-reactive antibodies, see the different IgG1 and IgG2a-2c rat immunoglobulins in this study), and contain complement-fixing antibody isotypes (IgG1 and IgG3 in humans [24], IgG2a and IgG2b in rats).

The mimotope-AQP4_268–285_-immunized animals produced AQP4-reactive antibodies in a titer range of 40–320, which at first glance seems rather low. However, such titers are frequently seen in NMOSD [69], where “low titer patients experience the same disease course as medium-titer and high-titer anti-AQP4 antibody patients” [70]. Low titers of AQP4-reactive antibodies could indicate their depletion from the circulation, for example by their binding to AQP4 expressing cells in the CNS [71] or in peripheral organs [72, 73]. However, we did not see any astrocyte-destructive, antibody-consuming lesions in the CNS (data not shown), had no evidence for AQP4 loss resulting from antibody binding to astrocytes in the area postrema (supplementary information S8), and could not detect AQP4 loss in kidney collecting duct epithelial cells which otherwise show most robust responses to AQP4-reactive antibodies [73] (data not shown). Hence, these AQP4-antibody seropositive Lewis rats might resemble human patients which can become AQP4-antibody positive months and decades prior to the onset of clinical NMOSD [74, 75].

The generation of AQP4-reactive antibodies in Lewis rats employed immunizations with mimotope-AQP4_268–285_. This immunogen replaces amino acid sequences of the conformational AQP4 epitope by a mimotope with completely different amino acid sequence, and brings this surrogate B cell epitope in close vicinity to T cell epitopes which are recognized by AQP4_268–285_-specific, highly encephalitogenic T cells present in the normal immune repertoire of wildtype Lewis rats [76]. Possibly, many different T cell epitopes would be equally efficient, provided that they drive strong T cell reactions, and are appropriately restricted by the major histocompatibility complex antigens of the immunized animals. B cell epitopes are often coupled to Keyhole Limpet Hemocyanin (KLH) or to Bovine Serum Albumin (BSA) for immunization. In all these cases, the immune system makes antibodies against the B cell epitope, and T cell responses against the carrier protein. Hence, the origin of B and T cell epitopes do not seem to matter for the antibody responses induced.

Immunizations with mimotope-T cell epitope fusion peptides have already been successfully employed in the past [77, 78], when it was noted that the presence of T cell epitopes in the fusion peptides positively affected B cell activation, levels of antibody production [77], and affinities of the induced antibodies [78].

Although our mimotope-AQP4_268–285_ fusions contained T cell epitopes for encephalitogenic AQP4_268–285_-specific T cells [76], we could not detect any T cell-induced inflammatory lesions in the CNS of the immunized animals (data not shown). It is possible that the time point for sacrificing the animals was too late to capture the window of T cell infiltration into the CNS. Alternatively, immunization with mimotope-AQP4_268–285_ could activate AQP4_268–285_-specific T cells in numbers sufficient to provide T cell help to B cells, but insufficient to overcome the threshold for the induction of CNS inflammation. This line of reasoning is supported by previous studies from our laboratory, were we could readily isolate AQP4_268–285_-specific T cells from AQP4_268–285_/CFA immunized Lewis rats, but were unable to induce experimental autoimmune encephalomyelitis by these immunizations [76].

Clearly, further strategies are required to improve our AQP4 autoantibody-producing animal model, in particular for studies of antibody entry into the CNS under physiological conditions in vivo, and of mechanisms causing relapse termination and tissue repair. These strategies may include the testing of additional mimotopes, the replacement of AQP4_268–285_ by other T cell epitopes, the use of other adjuvants, other routes of vaccine delivery, refined modes of boosting, and alternative time points for boosting, to elevate antibody titers and affinities, and to cause preferential production of antibodies with high complement-fixing abilities. However, our study provides proof of principle that the strategy to induce AQP4-reactive autoantibodies with mimotopes is feasible.

## Supplementary information

**Additional file 1.**

## Data Availability

The datasets used and/or analyzed during the current study are available from the corresponding author on reasonable request.
