# Supplementary material for: Induction of aquaporin 4-reactive antibodies in Lewis rats immunized with aquaporin 4 mimotopes
Source: Acta Neuropathol Commun. 2020 Apr 15;8:49. doi: 10.1186/s40478-020-00920-x (PMC7160927; doi:10.1186/s40478-020-00920-x)
Supplement: Supplementary file 1 — Additional file 1. [file 40478_2020_920_MOESM1_ESM.docx]

**S1 supplementary information.**

Identification of phages binding to NMO-IgG.

Briefly, Subcuvia^TM^ and the different NMO-IgGs used for mimotope search were diluted to 10 μg/ml in 0.1 M NaHCO3 (pH 8.6). 900 μl each of these dilutions were used to coat one well of a sterile 24-well plate (overnight, 4°C, 3 rpm on a shaker in a humidified chamber). After this step, the coating solution was replaced by blocking solution which was left in the wells for 2 hours at 4°C, while the plates were gently agitated at 3 rpm on a shaker in a humidified chamber.

We then continued with negative selection of unspecific phages using the Subcuvia^TM^-coated wells. The blocking solution was discarded from these wells, which were then washed six times with TBST 0.1% and incubated for 20 minutes at room temperature (RT) with 500 μl of the diluted 100-fold representation of the phage display library (1:50 in TBST 0.1%). During that time, phages binding unspecifically to immunoglobulins bound to Subcuvia^TM^ and were thus removed from the supernatant containing the remaining phages.

For positive selection of phages binding to AQP4-reactive antibodies in a given patient sample (i.e. in the NMO-IgG preparations I, II, III, or IV), we then transferred the supernatant after the negative selection onto wells coated with the relevant NMO-IgG, which had been washed immediately before six times with TBST 0.1%. Incubation was made for 40 minutes at RT and 3 rpm. During this time, NMO-IgG binding phages could adhere to the coated wells. After this incubation step, the supernatant was discarded, and the bound phages were collected from the wells using 500 μl elution buffer (10 minutes, room temperature, 3 rpm). The solution containing eluted phages was then transferred to a microtube and neutralized with 75 μl neutralization buffer (overnight, 4°C).

We then amplified phages from the neutralized eluate in E. coli. For this purpose, overnight cultures of the E. coli host strain ER2378 (37°C, 180 rpm, bacteria provided by the Ph.D.-12 library) were slowed down to 100 rpm for 15 min to allow the regeneration of the bacterial F-pili. These cultures were then diluted 1:100 in 20 ml LB+Tet medium, inoculated with the neutralized eluate, and incubated for 4.5 h at 37 °C and agitation at 225 rpm. During this step, phages grew within the bacteria and were released into the supernatant. Subsequently, the bacteria were pelleted by two consecutive centrifugation steps, the first using the complete bacterial suspension (12,000g, 10 minutes, 4°C), the second one using the supernatant of the first centrifugation step (12,000g, 10 minutes, 4°C). The phages were then precipitated from the supernatant (16 ml of the supernatant plus 2.6 ml PEG/NaCl incubated overnight at 4 °C), pelleted by centrifugation for 15 min at 4 °C and 12,000 g, washed in 1ml TBS, and collected after addition of 167 µl PEG/NaCl by centrifugation for 10 minutes at 4°C and 14,500g, 10 minutes, 4°C). The phages were then resuspended in 200 μl TBS and used for the next round of negative and positive selection on Subcuvia^TM^ and the NMO-IgG preparation used in the earlier selection process. In total, three rounds of negative/positive selection were made.

At the end of this selection series, the final amplified eluate was mixed with ER2738 and plated on IPTG/Xgal agar plates. Infected bacteria produced blue colonies, each of which contained the progeny of single phage clones. Individual colonies were picked and used for DNA sequencing.

**S2 supplementary information.**

Sequencing of phage clones to identify their displayed amino acid dodecamers

For DNA sequencing, single-stranded DNA was isolated from 98 phage clones which underwent negative and positive selection. 20 of these phage clones were selected using the NMO-IgG preparation I, 42 using the NMO-IgG preparation IV, 18 using the NMO-IgG preparation II, and 18 using the NMO-IgG preparaion III**.** 8 additional phage clones were randomly chosen from unselected phage display peptide library for further use as negative controls. The QIAprep® Spin M13 kit (Qiagen, Hilden, Germany) was used, essentially following the manufacturer´s instructions, and the DNA was sent to VBC-Biotech Service GmbH (Vienna, Austria) for sequencing with the 96 gIII sequencing primer. All data were checked for the presence of correct flanking sequences 3´and 5´to the mimotope DNA sequence to rule out faulty inserts. The DNA sequences were then translated into amino acid sequences using the ExPASy translate tool (web.expasy.org/translate). The scanner and reporter of target-unrelated peptides (SAROTUP, available at immunet.cn/sarotup) was used to exclude false positive hits. We could obtain the amino acid sequences of 62 different phage-displayed peptides (12 for I, 23 for IV, 15 for II, and 12 for III). 1 of these peptides was a possible plastic binder and therefore excluded from further studies. 4 peptides were found several times (IV-08 (5x), IV-12 (3x), IV-22 (2x), III-11 (2x)), and two peptides were retrieved from 2 different NMO-IgG preparations (I-18, identical to IV-08; I-07, identical to IV-04).

**S3 supplementary information**

ELISA with phage clones to identify binders to NMO-IgG

Single phage clones were used in ELISA to identify those binding best to “their” NMO-IgG (i.e. the NMO-IgG preparation used in the selections for these specific phages). BSA (the blocking agent used throughout the whole procedure) and human IgG (Subcuvia^TM^) served as negative controls. Wells of 96-well plates were coated in duplicates with 200 µl of NMO-IgGs and control human IgG (each with a concentration of 100 µg/ml in 0.1 M NaHCO3, or with 200µl of 0.1 M NaHCO3 which served as a control for the blocking reagent BSA later on. The plates were incubated overnight at 4°C. Then, the coating solutions were removed, and each well was blocked for 2 hours at 4°C with 300 µl 0.1 M NaHCO3/0.5% BSA. Then, the blocking solution was discarded and the plates were washed six times with 50mM Tris-HCl (pH 7.5)/150 mM NaCl/0.5% [v/v] Tween-20. Then, 100 µl 50mM Tris-HCl (pH7.5)/150 mM Nacl/0.1% [v/v] Tween-20 containing 10^8^ phages were added to each well, and the plates were incubated for 1 hour at 4°C on a rotating platform (3rpm). Afterwards, the unbound phages were removed and the plates washed six times with 50mM Tris-HCl (pH 7.5)/150 mM NaCl/0.5% [v/v] Tween-20. Horseradish peroxidase-conjugated anti-M13 monoclonal antibodies (1:5000 in 0.1 M NaHCO3/0.5% BSA) were added in a volume of 200 µl to each well, and incubated for 1 hour at room temperature with gentle agitation (3rpm). After 6 additional washing steps with 50mM Tris-HCl (pH 7.5)/150 mM NaCl/0.5% [v/v] Tween-20, 100 µl 3,3´,5,5´-tetramethyl-benzidine liquid substrate (TMB; from Sigma) were applied to each well and incubated for 15 min at room temperature, protected from light. Finally, 100µl Stop reagent for TMB substrate (Sigma) were added to each well and the plates were read with a microplate reader (GloMax®-Multi Detection System, Promega, Madison WI, USA) at 450 nm.

These ELISAs lead to the identification of 6 phage clones from the I biopanning series, 13 phage clones from the IV biopanning series, and 4 phage clones from the II biopanning series.

Since we did not keep phages from the biopannings with III, we used the mimotope mapping tool PepSurf ([1], available from http://pepitope.tau.ac.il/sources.html) to screen the peptides predicted by the DNA sequences for those predicted to map to the extracellular loops of human AQP4 (protein data base (PDB) of the Research Collaboratory for Structural Bioinformatics (RCSB), accession number 3GD8). This led to the selection of 4 peptides from the III biopanning series (fig 2).

**S4 supplementary information**

Antibody blocking assays

Since mimotopes bind to the antigen recognition sites of an antibody, pre-incubation of AQP4-reactive antibodies with mimotopes should interfere with antibody binding to AQP4-transfected human embryonic kidney (HEK) cells.

Peptides used for flow cytometry

12-mer amino acid sequences, C-terminally attached to the amino acids GGGS were synthesized by Centic Biotec (Heidelberg, Germany), with or without N-terminal acetylation and C-terminal amidation. We did not observe any differences between the different peptides containing these additional modifications or not, and used both of them without further discrimination.

Additional 12-mer peptides C-terminally attached to the GGGS linker were synthesized by JPT Peptide Technologies GmbH (Berlin, Germany).

Cells

HEK293A cells were transiently transfected with an expression vector containing the sequence of a C-terminal human AQP4(M23)-emGFP fusion protein under control of the human cytomegalovirus promoter (pcDNA6.2/C-emGFP-Topo-hAQP4(M23) [2], essentially as described [2]. 24-48 hrs after the transfection, the cells were harvested by trypsinization, and incubated for 30 min at 4°C on a rotating platform (10rpm) in blocking solution (1xPBS/1mM EDTA/10% goat serum (heat-inactivated at 56°C for 30 min)/0.02 mg/ml goat IgG). Then, the cells were centrifuged (10 min/400g, 4°C) and resuspended in FACS-buffer (1xPBS/1mM EDTA/10% heat-inactivated fetal bovine serum) in a density of 1-2x10^6^ cells/ml. 200 µl of this suspension was then added to a 96-well plate and centrifuged (5 min/400g, 4°C). The supernatant was removed. The pellets were resuspended in 100 µl FACS-buffer (“unstained control”), in FACS-buffer containing NMO-IgG plasmapheresate (stock 10mg/ml, diluted 1:100-1:200 to allow for target cell staining in the linear range of antibody binding; “NMO-IgG only”, from 15 different NMO-IgG preparations), in FACS-buffer containing the same NMO-IgG incubated before with a 500-fold excess of peptides, or in FACS-buffer containing human control IgG (Subcuvia^TM^, stock 10mg/ml, diluted 1:100 - 1:200), and incubated for 30 min at 4°C on a rotating platform (10rpm). All stainings were done in duplicates or triplicates.

Afterwards, the cells were washed three times with 100µl FACS-buffer, and incubated for 30 min at 4°C on a rotating platform (10rpm) in 100µl FACS-buffer containing goat anti-human Cy3 (Jackson ImmunoResearch Laboratories, 1:200). Subsequently, the cells were washed three times with 100µl FACS-buffer, resuspended in 100µl FACS-buffer containing 7-amino-actinomycin D (7-AAD, 1:200, eBioscience Inc, San Diego, USA) for the discrimination of live and dead cells, and brought to a final volume of 300µl with FACS-buffer.

Flow cytometric analysis was conducted at the Core Facility Flow Cytometry of the Medical University of Vienna, using a BD LSRFortessa^TM^ cell analyzer. Data were acquired and analyzed with the software BD FACSDiva 6.1.3.

**S5 Supplementary information**

**Staining of rat astrocytes with patient-derived IgG**

Astrocyte-enriched mixed glia cultures were cultured in PLL-coated 96-well plates and were used in triplicate wells. In a first step, the cells were washed 3 x with ice-cold RPMI medium, and incubated with 100 µl IgGs (stock 10 mg/ml, diluted 1:1000) of 4 different AQP4 antibody-positive NMO patients (NMO-IgG I – IV), 1 AQP4 antibody-negative NMO patient (negative control), or 1 AQP4 antibody-negative MS patient (negative control) for 30 min at 4°C. The cells were washed 3 x with ice-cold RPMI medium. All following steps were then made in the dark. 100 µl goat-anti-human-Cy3 (diluted 1:100 in 10%FCS/RPMI medium) were applied for 45 min at 4°C, the cells washed 3x with 1xPBS and subsequently fixed with 100 µl 4% paraformaldehyde for 15 min at room temperature. After an additional washing step (3x with 1xPBS), the cells were permeabilized with 0.1% Triton-X-100 in 10% FCS/RPMI for 5 min at room temperature, and washed again 3x with 1xPBS. Then, 100 µl of a mouse monoclonal anti-GFAP antibody (Neomarkers, 1:100 in 10% DAKO diluent/PBS) was applied (overnight, 4°C). The cells were washed 3 x with 1xPBS, incubated with 100 µl donkey-anti-mouse-Cy2 (1:100 in 10% FCS/DAKO buffer) for 1 hour at room temperature, and then washed for a last time 3x with 1xPBS.

**
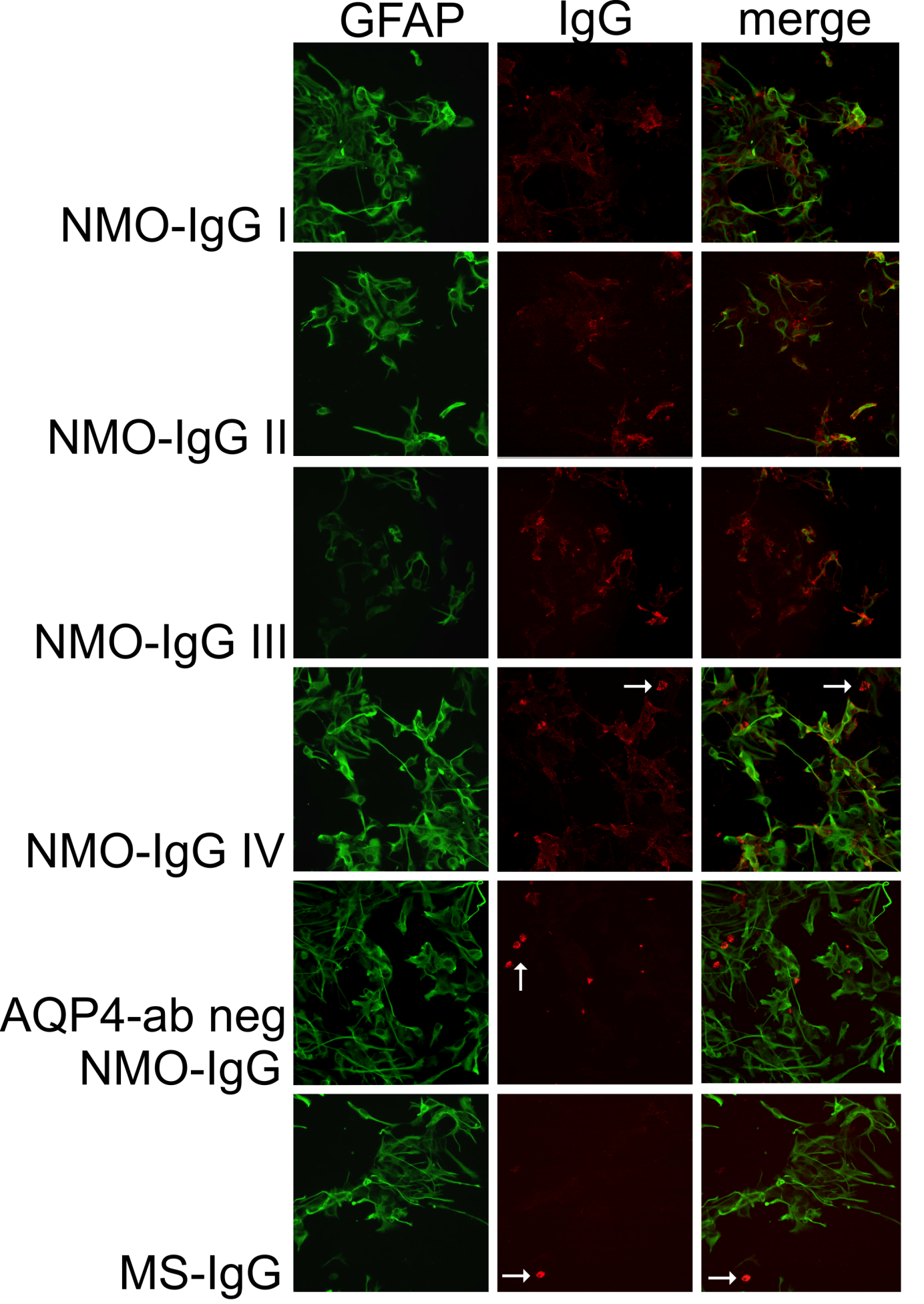
**

**Fig. legend:**

Immunoglobulin preparations of 4 NMO patients (NMO-IgG I – NMO-IgG IV), of 1 AQP4-antibody negative NMO patient (AQP4-ab neg NMO-IgG), and of 1 patient with multiple sclerosis (MS-IgG1) were used for live staining of astrocytes *in vitro*. Bound AQP4-specific antibodies were visualized with anti-human Cy3 (“IgG”, red). After fixation and permeabilization of the cells, monoclonal antibodies specific for GFAP were applied and visualized with anti-mouse Cy2 (green). Please note binding of AQP4-specific antibodies from the preparations NMO-IgG I – NMO-IgG IV, and an absence of AQP4-specific antibodies in the control preparations (AQP4-ab neg NMO-IgG, MS-IgG I).

The white arrows point to contaminating microglial cells, which unspecifically bound antibodies used in live cell-stainings. Pictures were adjusted for intensity and brightness to make the positive staining results better visible

**S6 Supplementary information**

Astrocyte-enriched mixed glia cultures were cultured in PLL-coated 96-well plates and were used in triplicate wells. In a first step, the cells were washed 3 x with ice-cold RPMI medium, and subsequently exposed for 30 min at 4°C to 100 µl human control IgG (Subcuvia^TM^, 10mg/ml, diluted 1:1000). This step was made to saturate the Fc receptors of contaminating microglia cells as much as possible. Next, the cells were washed again 3x with ice-cold RPMI, and incubated with 100 µl undiluted serum of mimotope-AQP4_268-285_/CFA immunized Lewis rats for 30 min at 4°C. The cells were washed 3 x with ice-cold RPMI medium. All following steps were then made in the dark. 100 µl goat-anti-rat-Cy3 (diluted 1:100 in 10%FCS/RPMI medium) were applied for 45 min at 4°C, the cells washed 3x with 1xPBS and subsequently fixed with 100 µl 4% paraformaldehyde for 15 min at room temperature. After an additional washing step (3x with 1xPBS), the cells were permeabilized with 0.1% Triton-X-100 in 10% FCS/RPMI for 5 min at room temperature, and washed again 3x with 1xPBS. Then, 100 µl of a polyclonal rabbit anti-GFAP antibody (Dako, 1:500 in 10% DAKO diluent/PBS) was applied (overnight, 4°C). The cells were washed 3 x with 1xPBS, incubated with 100 µl donkey-anti-rabbit-Cy2 (1:100 in 10% FCS/DAKO buffer) for 1 hour at room temperature, and then washed for a last time 3x with 1xPBS.

**
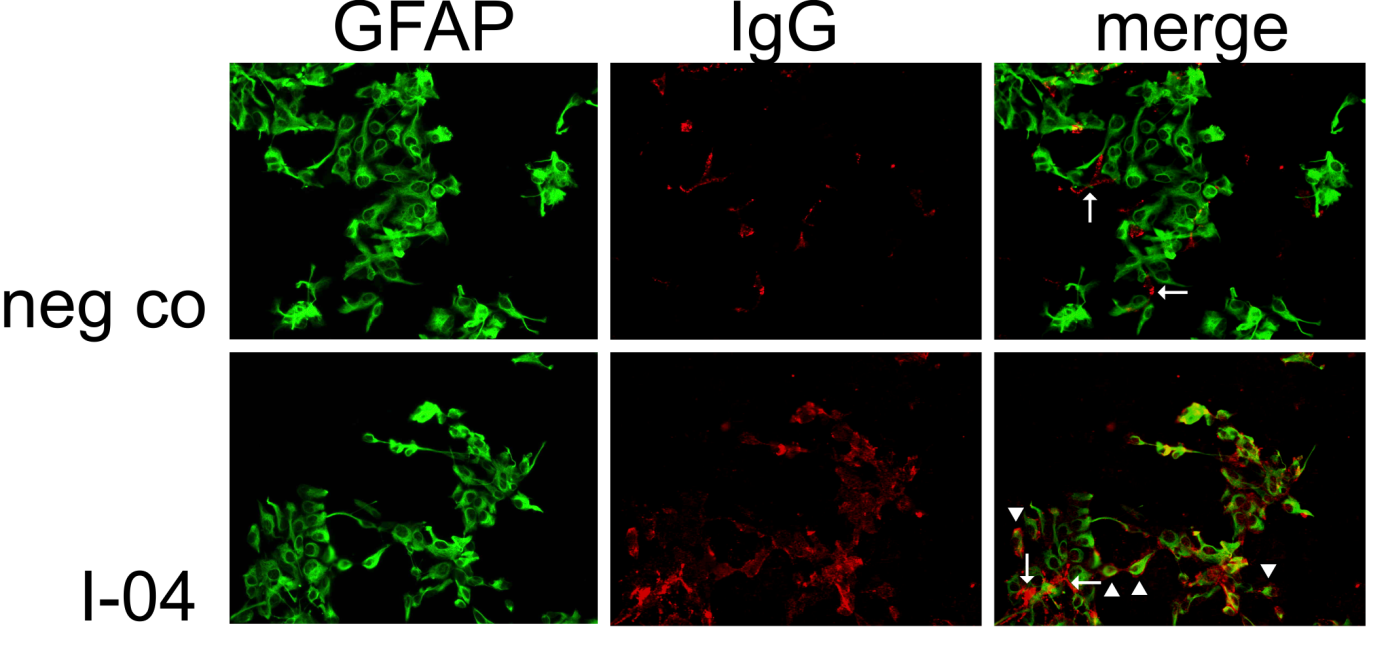
**

**Fig. legend:**

Astrocyte-enriched mixed glia cultures were exposed to serum of a III-01-AQP4_268-285_/CFA immunized Lewis rat with an anti-rat AQP4 antibody titer of 0 (negative control, animal identified in table 3) or serum of a I-04-AQP4_268-285_/CFA immunized Lewis rat with an anti-rat AQP4 antibody titer of 320 (animal identified in table 3). Bound antibodies were then reacted with goat anti-rat Cy3 (red). After fixation and permeabilization of the cells, polyclonal antibodies specific for GFAP were applied and visualized with anti-rabbit Cy2 (green). The white arrows point to contaminating microglial cells, which unspecifically bound antibodies used in live cell-stainings, the white arrow heads show double-stained astrocytes.Pictures were adjusted for intensity and brightness to make the positive staining results better visible

**7**

**S8 supplementary information**

Any attempts to detect bound AQP4-reactive rat antibodies on rat target cells are hampered by the presence of irrelevant rat antibodies in the tissue, since the secondary antibodies used in immunohistochemistry will bind to both types of antibodies alike. To avoid these technical problems, we used a different read-out for the presence/absence of AQP4-antibody binding: the AQP4-reactive antibody-induced loss of AQP4 in astrocytes of the area postrema, and in collecting duct epithelial cells of the kidney [3].

Immunohistochemistry

2–4 μm thick sections of area postremae were cut on a microtome. The sections were dewaxed in xylol for 30 min, transferred to 96% ethanol, and incubated in 0.2% hydrogen peroxide for 30 min to block endogenous peroxidase. Then, the sections were rehydrated through a descending ethanol series (96, 70, 50%), rinsed in distilled water, and subjected to antigen retrieval by heating them for 60 minutes in 10 mM EDTA pH 8.5 in a conventional household steamer. Subsequently, the sections were rinsed in 0.1 M PBS or Tris-buffered saline (TBS) for 60 min, and exposed to 10% fetal calf serum (FCS) in 1 x DAKO Wash Buffer in PBS for 20 min at room temperature to reduce non-specific background. Then, immunohistochemical stainings were done essentially as described [4], using polyclonal rabbit anti-rat AQP4 (1:250, Sigma-Aldrich, Vienna, Austria). Immunohistochemistry was completed by incubation with biotinylated donkey anti-rabbit antibodies (1:2000, Jackson ImmunoResearch) followed by exposure to avidin-peroxidase complex (1:100 in DB/FCS; Sigma). Labeling was visualized with 3,3’ diaminobenzidine-tetra-hydrochloride (DAB, Sigma) containing 0,01% hydrogen peroxide. All sections were counterstained with Meyer’s hematoxylin, dehydrated and mounted in Eukitt^©^ (Merck, Darmstadt, Germany).

**Absence of antibody-induced changes in the area postrema**

We compared the AQP4 expression patterns in the area postremae of mimotope-AQP4_268-285_-immunized animals producing AQP4 antibodies (titers ≥ 40, n=6) or not (titer = 0, n=1; titer ≤ 20, n=1) with those seen in untreated Lewis rats (n=2) or in Lewis rats challenged i.p. for 48 hrs (n=5) or 120 hrs (n=5) with mouse control IgG (the mouse IgG-challenged tissue derived from our archive and was extensively described [3]). We found that these patterns were all in the same range (S5 fig), indicating that the amounts of AQP4-reactive antibodies binding to the astrocytes were too low to cause AQP4 loss, and hence too low to deplete the antibodies from the serum.

**
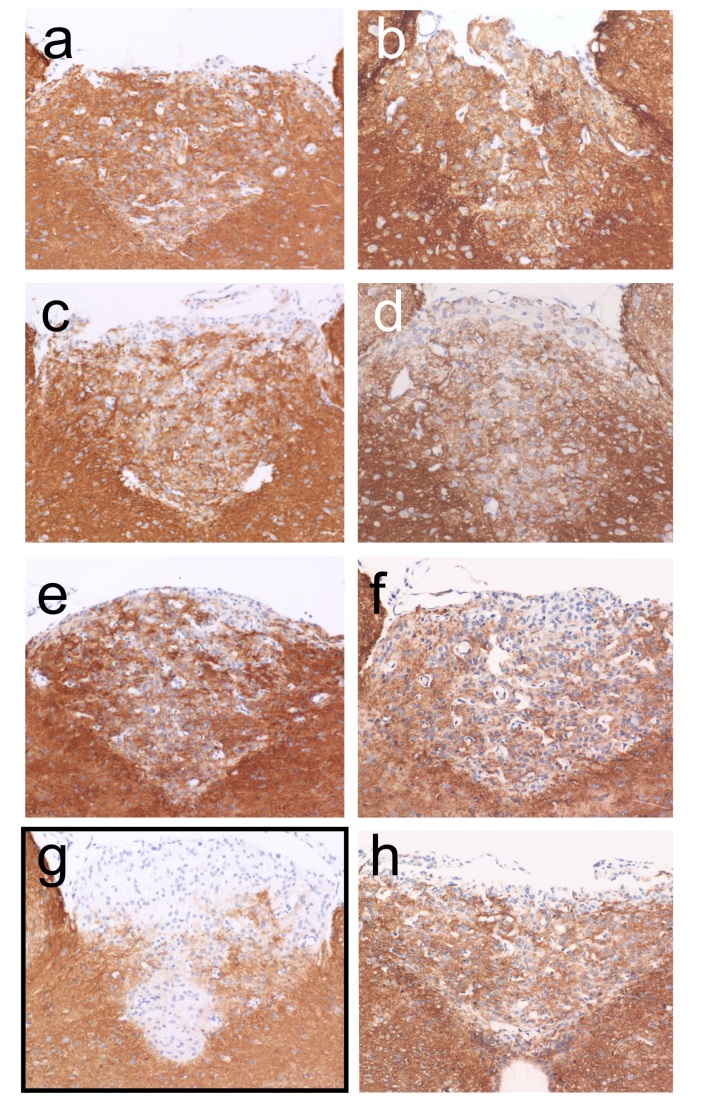
**

**Fig. legend: Area postremae of Lewis rats stained with antibodies against intracellular AQP4 epitopes to reveal heterogenous AQP4 expression at this site, both in mimotope-AQP4_268-285_ immunized animals and in controls.**

Shown here is an AQP4 staining pattern as representative for untreated Lewis rats (n=2) and Lewis rats which were seropositive for mouse IgG for 120 hours (a, n=5) or 48 hours (c and e, n=5). Also shown is the AQP4 reactivity of the tissue from animals which were seropositive for 24 hours for the monoclonal anti-AQP4 antibody E5415A [3] (g, n=5, positive control for AQP4 loss in response to anti-AQP4 antibodies), or have been immunized with mimotope-AQP4_268-285_ and became anti-AQP4 antibody-reactive. The immunogens were I-13-AQP4_268-285_ (titer 1:320, b), I-13-AQP4_268-285_ (titer 1:160, d), III-01-AQP4_268-285_ (titer 1:80, f), and IV-04-AQP4_268-285_ (titer 1:80).

**Supplementary references**

1. Mayrose I, Shlomi T, Rubinstein ND, Gershoni JM, Ruppin E, Sharan R, Pupko T: Epitope mapping using combinatorial phage-display libraries: a graph-based algorithm. Nucleic Acids Res 2007, 35: 69-78.

2. et Mader S, Lutterotti A, Di Pauli F, Kuenz B, Schanda K, Aboul-Enein F, Khalil M, Storch MK, Jarius S, Kristoferitsch W, Berger T, Reindl M: Patterns of antibody binding to aquaporin-4 isoforms in neuromyelitis optica. PLoS One 2010, 5: e10455.

3. Hillebrand S, Schanda K, Nigritinou M, Tsymala I, Bohm D, Peschl P, Takai Y, Fujihara K, Nakashima I, Misu T, Reindl M, Lassmann H, Bradl M: Circulating AQP4-specific auto-antibodies alone can induce neuromyelitis optica spectrum disorder in the rat. Acta Neuropathol 2019, 137: 467-485.

4. Bradl M, Misu T, Takahashi T, Watanabe M, Mader S, Reindl M, Adzemovic M, Bauer J, Berger T, Fujihara K, Itoyama Y, Lassmann H: Neuromyelitis optica: pathogenicity of patient immunoglobulin in vivo. Ann Neurol 2009, 66: 630-643.
